# WDR72 Gene Variant Associated With Distal Renal Tubular Acidosis, Enuresis, Enamel Hypoplasia, Renal Cysts, and Renal Calculi: A Case Report

**DOI:** 10.7759/cureus.87894

**Published:** 2025-07-14

**Authors:** Anwar AL-Omairi, Abdullah Alabbas, Tasneim Makki, Suliaman Al Saidi, Mohammed Alriyami

**Affiliations:** 1 Pediatric Nephrology, Sultan Qaboos University Hospital, Muscat, OMN; 2 Pediatric Nephrology, American Hospital Dubai, Dubai, ARE; 3 Pediatrics, Sultan Qaboos University Hospital, Muscat, OMN; 4 Pediatric Nephrology, The Royal Hospital, Muscat, OMN

**Keywords:** amelogenesis imperfecta (agi), dental enamel hypoplasia, distal renal tubular acidosis, nephrocalcinosis (nc), renal calculi, renal cyst, wdr72 gene mutation

## Abstract

Amelogenesis imperfecta IIA3, caused by mutations in the tryptophan-aspartate repeat domain 72 (*WDR72*) gene, has recently been linked to distal renal tubular acidosis (dRTA). This genetic cause of dRTA has been rarely reported, and its full phenotypic spectrum is still being explored. This case report aims to share the clinical presentation and genetic findings of a recently encountered patient with this genetic variant. An eight-year-old girl presented with nocturnal enuresis and enamel hypoplasia. Laboratory investigations revealed normal anion gap metabolic acidosis with inappropriately high urine pH, along with nephrocalcinosis, renal calculi, and a renal cyst. Genetic testing confirmed the presence of a variant in the *WDR72* gene. In addition to the known complications of dRTA, such as nephrocalcinosis and renal calculi, this variant might also be associated with renal cysts. This case adds to the limited literature by suggesting a possible association between *WDR72* variants and renal cysts, an uncommon finding that may expand the phenotypic spectrum of this condition.

## Introduction

Amelogenesis imperfecta IIA3 is a rare autosomal recessive condition caused by mutations in the tryptophan-aspartate repeat domain 72 (*WDR72*) gene. Recently, it has been linked to distal renal tubular acidosis (dRTA) [1,2]. The genetic link between the two conditions has been reported from different parts of the world; however, little is known about the pathophysiology and the phenotype of this unique mutation that causes dRTA [3]. This case report aims to share the clinical presentation and genetic findings of a recently encountered patient.

## Case presentation

An eight-year-old girl presented with a history of polyuria and nocturnal enuresis. She had no history of dysuria, flank pain, urinary tract infections, or diarrhea, and her bowel habits were normal. Her medical history was notable for severe dental issues, including orange-brown stained teeth and multiple dental caries. She was not taking any regular medication, and her family history was unremarkable, except for two maternal cousins with similar dental problems. There was no consanguinity between the parents. The affected cousins were not evaluated for similar renal or metabolic abnormalities.

On examination, her blood pressure was 102/76 mmHg, which lies between the 50th and 90th centiles, with normal skin perfusion, and she was neither pale nor jaundiced. Her weight was tracking along the fifth percentile, and height along the 10th percentile. Cardiopulmonary examination was unremarkable, with clear lungs and normal heart sounds. Her abdomen was soft, with no organomegaly, and her kidneys were not palpable. Vision and hearing were normal.

**Figure 1:**
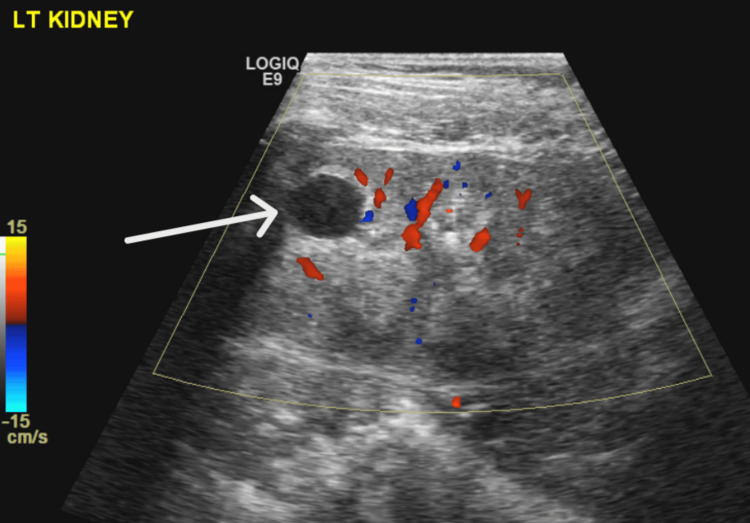
Left kidney simple renal cyst

**Figure 2:**
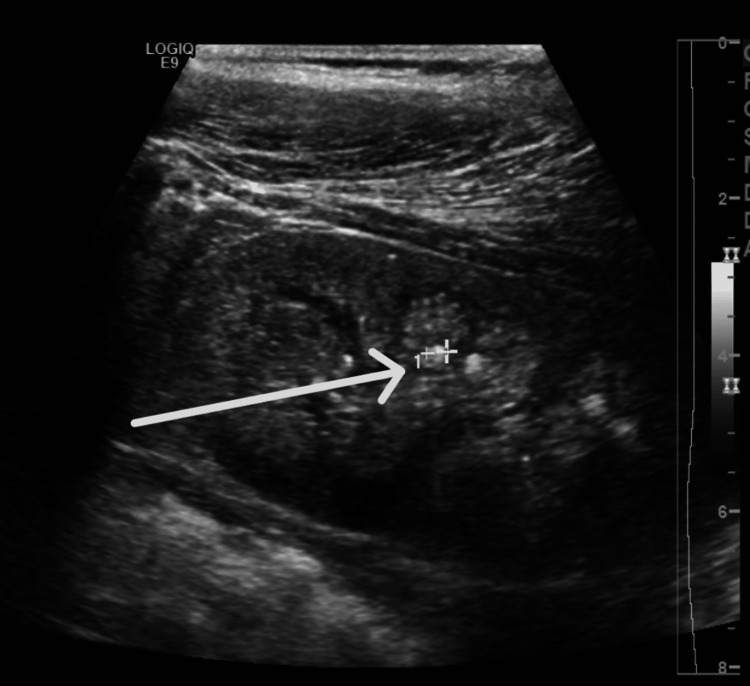
Renal stone and nephrocalcinosis

Genetic testing using a comprehensive nephrology gene panel revealed a homozygous frameshift variant in the *WDR72* gene: c.2857del p.(Ser953Valfs*20). No pathogenic or likely pathogenic variants were identified in other genes known to be associated with renal tubular acidosis. Renal ultrasound showed bilateral medullary nephrocalcinosis and multiple small non-obstructive renal calculi. There was a simple renal cyst seen in the upper pole of the left kidney (Figures 1, 2). Relevant biochemical and urine investigations are summarized in Tables 1, 2.

**Table 1:** Blood biochemistry at initial presentation Co_2_: carbon dioxide; *arterial blood gas reference range

Test	Patient Value	Reference Range
Sodium	139 mmol/L	135-145 mmol/L
Potassium	3.6 mmol/L	3.5-5.1 mmol/L
Chloride	110 mmol/L	98-107 mmol/L
Urea	2.9 mmol/L	2.8-8.1 mmol/L
Phosphate	1.61 mmol/L	1.05-1.7 mmol/L
Alkaline phosphatase	392 u/L	129-417 u/L
Creatinine	45 µmol/L	30-47 µmol/L
Anion gap	11 mmol	5-14 mmol
pH	7.21	7.35 - 7.45
CO_2_	47.9 mmHg	32-45 mmHg*
Bicarbonate	16.4 mmol/L	21.8-26.9 mmol/L
Lactate	1.1 mmol/L	0.5-1.6 mmol/L
Calcium ionized	1.28 mmol/L	1.15- 1.29 mmol/L

**Table 2:** Urine electrolytes and dipstick *Urine electrolytes are interpreted based on clinical context.

Test*	Patient Value
Urine Sodium	52 mmol/L
Urine Potassium	33 mmol/L
Urine Chloride	55 mmol/L
Urine Anion Gap	30 mmol
Urine pH	7
Urine Glucose	Nil

Laboratory evaluation revealed normal anion gap metabolic acidosis (NAGMA), nephrocalcinosis, and impaired urine acidification. Serum phosphate and alkaline phosphatase levels were within normal limits, and there was no proteinuria or glucosuria to suggest Fanconi syndrome. These findings were consistent with a diagnosis of dRTA. The patient was treated with a prolonged-release granule of potassium citrate and potassium hydrogen carbonate 24 mEq twice daily. Follow-up lab work revealed normal blood gas. 

## Discussion

This patient exhibited severe dental disease known as amelogenesis imperfecta IIA3, caused by a mutation in the *WDR72* gene. She also presented with NAGMA and nephrocalcinosis on ultrasound, indicating dRTA [4,5]. dRTA is characterized by a defect in type A intercalated cells, leading to impaired acid excretion in the urine. It is typically diagnosed by the presence of NAGMA and evidence of impaired renal acid excretion. The distal renal tubule secretes H+, which is buffered by ammonia to form ammonium. Therefore, the ammonium level in urine is the best indicator for acid excretion; however, testing urine ammonium is technically difficult and not widely available. Checking urine pH and urine anion gap are indirect tools to assess acid excretion. Urine pH more than 5.5 in the presence of acidemia is considered abnormal. The urine anion gap is based on the concept that anions and cations are balanced in the urine. The main cations, sodium, potassium, and ammonium, should be roughly equal to the main anion, chloride. Unlike ammonium, sodium, potassium, and chloride, urine tests are readily available [6]. This patient had a urine pH and a urine anion gap of 30 mmol, which clearly indicated impaired urine acidification. Adding to that, the presence of renal stone and nephrocalcinosis is a well-known complication and feature of dRTA [7,8].

The association between *WDR72* mutations and dRTA was first reported by Rungroj et al. in 2018, and subsequent reports have highlighted this connection [9]. As of 2023, 14 cases of amelogenesis imperfecta due to *WDR72 *gene variants have been reported in association with distal renal tubular acidosis [10].

The *WDR72* gene is expressed in intercalated cells, and while it is believed to play a role in intracellular trafficking, the precise mechanism by which mutations in this gene cause dRTA remains unclear. The severity of dRTA due to *WDR72* mutations appears to vary, with many cases presenting mildly [11]. In this patient, the disease was relatively mild but associated with enuresis, renal calculi, and nephrocalcinosis, all of which are common complications of dRTA [3]. Additionally, the patient had a well-defined cortical renal cyst in the upper pole of the left kidney, measuring approximately 1.3 cm x 1.1 cm x 1.1 cm. No family history of cystic kidney disease was noted, and genetic testing did not reveal any relevant mutations associated with cystic kidney disease. The association between renal cyst and this variant can not be generalized based on a single case report. Renal cysts have not been previously reported in cases with *WDR72* mutations, though they have been associated with other causes of dRTA. 

## Conclusions

Mutations in the *WDR72* gene are increasingly recognized as a cause of both dRTA and amelogenesis imperfecta IIA3, representing a syndromic presentation that affects both dental and renal systems. While the association with enamel defects and impaired acid-base homeostasis is well-established, emerging evidence from this case suggests that *WDR72* mutations may also predispose patients to additional renal manifestations, such as the development of renal cysts. This expanded phenotype highlights the importance of comprehensive renal evaluation in patients with *WDR72*-related disorders and supports the need for long-term monitoring to better understand the natural history and potential complications of this rare genetic condition.
